# Frequency and pattern of congenital heart defects in a tertiary care cardiac hospital of Karachi

**DOI:** 10.12669/pjms.321.9029

**Published:** 2016

**Authors:** Najma Pate, Shama Jawed, Nagina Nigar, Fariha Junaid, Asia Abdul Wadood, Fatima Abdullah

**Affiliations:** 1 Najma Patel, MBBS, FCPS, FSCAI. Associate Professor and Head of the Pediatric Cardiology Department, National Institute of Cardiovascular Diseases, Karachi, Pakistan; 2 Shama Jawed, MBBS, Dow University of Health Sciences, Karachi, Pakistan; 3 Nagina Nigar, MBBS, Jinnah Sindh Medical University, Karachi, Pakistan; 4 Fariha Junaid, 5^th^ year MBBS student of Dow University of Health Sciences, Karachi, Pakistan; 5 Asia Abdul Wadood, MBBS, Jinnah Sindh Medical University, Karachi, Pakistan; 6 Fatima Abdullah, MBBS, Jinnah Sindh Medical University, Karachi, Pakistan

**Keywords:** Congenital heart defects, Ventricular septal defects, Tetralogy of fallot, Cyanotic heart defect

## Abstract

**Objective:**

To determine the current frequency and pattern of distribution of congenital heart defects (CHD) at National Institute of Cardiovascular Diseases (NICVD), with the age at which initial diagnosis of CHD was made and the age at which the participant first visited the study center.

**Methods:**

This is a descriptive and prospective hospital based study conducted in the pediatric cardiology unit outpatient department of NICVD. This study included all the patients, irrespective of age, having confirmed diagnosis of CHD on the basis of echocardiographic report. The collected data was entered and analyzed by using Statistical Package for Social Sciences v 20.0.

**Results:**

Out of 1100 cases of congenital heart defects 1003 could be analyzed. There are 565 males (56.3%) and 438 females (43.6%). Total 609 cases (60.6%) were of simple acyanotic lesions and 387 (38.6%) were complex cyanotic lesions. In simple lesions septal defects constitute 64.9% and obstructive lesions were 11.0%. Tetralogy of fallot(TOF) was the commonest CHD and cyanotic lesion accounted for 24.4% of the total 1003 cases followed by Ventricular septal defect (VSD) 21.5%, Atrial septal defect (ASD) 9.3% and Patent ductus arteriosus (PDA) 8.6%. Pulmonary stenosis(PS) was the most common obstructive lesion making 3.1% of the CHD. In 147 (14.5%) cases combination of simple defects were encountered and the commonest combination was ASD with VSD in 34 cases.

**Conclusion:**

Congenital Heart Defects are very common in our setup and early detection of CHD is increasing. Overall burden of CHD is also increasing therefore a proper population based study on a large scale is needed to estimate the prevalence accurately.

## INTRODUCTION

Congenital heart defect is defined as structural malformation of heart or great vessels that is present by birth. It includes the defects in the interior walls of the heart, septation of the chambers and their sequence, the valves inside the heart and/or the arteries and veins. There are different types of congenital heart defects which can be broadly classified as simple defects and complex defects. It is the most common single group of congenital abnormalities accounting for about 30 percent of the total and has high mortality rate during infancy, depending on the type and severity of lesion.^1^-^3^ It is necessary to mention that the heart ailment is a defect or abnormality, not a disease.^4^ Congenital heart defects can be found as either an isolated lesion or in combination with other heart defects. These are also found as a part of/in association with certain syndromes.^3^ Some types of CHD are grouped into Major congenital heart defect (MCHD) defined as a complex structural malformation of the heart and/or the great arteries, and/or the presence of a defect requiring surgical or catheter intervention within the first 6 months of life.^5^

The incidence of CHD is different in studies conducted in different countries. **In USA the studies published after 1955 reports incidence ranging from 4-50/1000 live births and it accounts for 3% of all infant deaths.^6^,^7^ The Baltimore-Washington infant study reports the rate of 4/1000 live births of CHD.^8^** A systematic review and meta-analysis report showed that the highest total birth prevalence of CHD is 9.3/1000 live birth and it is of Asia.^9^ In developing countries the burden of congenital heart defects is increasing day by day because of increase in risk factors and etiological factors for these defects, like older age of mothers, infections during pregnancies etc. Occurrence of CHD in developing country like ours, would be much higher despite of the fact that most of the cases in our setup are missed due to lack of proper facilities, detection modalities and diagnostic techniques. Moreover most of the cases present late in a cardiac center, when complications have already developed because of delay in the diagnosis of CHD. This adds further to the mortality rate of CHD which is already high. In developed countries early detection and proper treatment has increased the survival rate and has decreased mortality from 80% to 20% causing an increase in the number of adults with CHD.^10^

Although few hospital based studies have been conducted at the regional level to show the prevalence of CHD, unfortunately data of the incidence or prevalence at national level that can show the burden of congenital heart defect in our country, is not available. Moreover, there is no registration system at national level to estimate the total number of cases. The age at which such patients are initially diagnosed is also not known. Most of the studies considered the age at which, case of CHD visit the center of study as the age at which initial diagnosis is made.

Thus this study was conducted in a tertiary care cardiac hospital, National Institute of Cardiovascular Diseases with the aim to determine the current pattern of distribution of CHD in the population and the age at which initial diagnosis of CHD was made along with the age at which the participant first visited the study center.

## METHODS

This is a descriptive and prospective hospital based study in which patients visiting NICVD pediatric outpatient department (OPD) for follow up were enrolled based on the following inclusion and exclusion criteria.

### Inclusion criteria

Patient can be of any age and must be a diagnosed case of congenital heart defect on the basis of ECHO done in NICVD.

### Exclusion criteria

Patient having unconfirmed diagnosis of CHD or diagnosed case of Acquired Heart Disease.

Our aim was to collect at least data of 1000 cases of CHD in our defined study period of two months covering around 20 OPD days, using a properly designed data sheet. The required information was entered in the sheet by interviewing the study participants and from their hospital record. Duplication of the data was avoided by entering the hospital registration number. Total 1100 subjects were enrolled and data sheets were filled, of which only 1003 were analyzable. Collected data was entered and analyzed by using Statistical Package for Social Sciences v 20.0 (SPSS, Inc., Chicago, IL, USA). Descriptive statistics including frequencies, mean and percentages are calculated.

## RESULTS

Out of 1100 cases of congenital heart defects 1003 could be analyzed. There were 565 males (56.3%) and 438 females (43.6%). They were divided into different age groups to identify the age group at which initial diagnosis of CHD and 1^st^ visit at NICVD was made. In majority of cases, 74.7%, initial diagnosis was made during infancy. In 59.3% of cases first visit to NICVD was done during infancy (Table-I).

**Table-I:** Age at the time of Initial diagnosis and 1st visit to study center.

Age groups	Age at the time of initial diagnosis		Age at the time of 1^st^ visit to NICVD	

	Male	Female	Male	Female
0-1 Year	427	323	331	264
>1-2 Years	42	33	72	41
>2-5 Years	50	42	62	55
>5-8 Years	14	12	38	24
>8-11 Years	13	6	28	20
>11-15 Years	11	8	25	17
>15-20 Years	4	5	5	6
>20-25 Years	2	2	2	3
>25 Years	2	7	2	8

Out of 1003 cases of CHD, 609 cases (60.6%) were of simple acyanotic lesions and 387 (38.6%) were complex cyanotic lesions. In simple lesions septal defects constitute 64.9% and obstructive lesions were 11.0%. TOF was the commonest CHD and cyanotic lesion constituting 24.4% of the total 1003 cases followed by VSD 21.5%, ASD 9.3% and PDA 8.6%. Pulmonary stenosis was the most common obstructive lesion making 3.1% of the CHD followed by Aortic stenosis 1.89% and Coarctation of aorta 1%. Three cases of hypertrophic cardiomyopathy, 1 case of sub-aortic stenosis and congenital WPW syndrome have also been recorded. (Table-II)

**Table-II:** Pattern of Congenital Heart Defects.

Type of defect	Subtype		Number of cases (n)	Percentage (%)
Simple Defects n = 609 (60.7%)	Septal n=395 (65.1%)	VSD	216	21.5
		ASD	93	9.3
		PDA	86	8.6
	Obstructive n = 67 (11%)	Pulmonary Stenosis	31	3.1
		Aortic Stenosis	19	1.89
		Coarctation of aorta	10	1
		Cong. Mitral Stenosis	3	0.3
		Cong. Mitral Regurgitation	4	0.4
	Combinations of simple defect n = 147 (24.1%)	Two in combination	129	12.7
		More than 2	18	1.8
Complex or major congenital heart defects n = 387 (38.6%)	Tetralogy of fallot (63.3%)		245	24.4
	Single ventricle (13.4%)		52	5.2
	Transposition of great arteries (12.7%)	d-TGA	41	4.1
		l-TGA	8	0.8
	AV Canal Defect (5.9%)		23	2.3
	Total anomoulus pulmonary venous return (4.1%)		16	1.6
	Truncus arteriosus (0.5%)		2	0.2
Misc n = 7	Hypertrophic cardiomyopathy		3	0.3
	Bicuspid aortic valve		2	0.2
	Congenital WPW Syndrome		1	0.1
	Sub-aortic stenosis		1	0.1

Total	1003	100%		

VSD: ventricular septal defect, ASD: Atrial septal defect, PDA: Patent ductus arteriosus, AV: Atrioventricular

**Fig.1:**
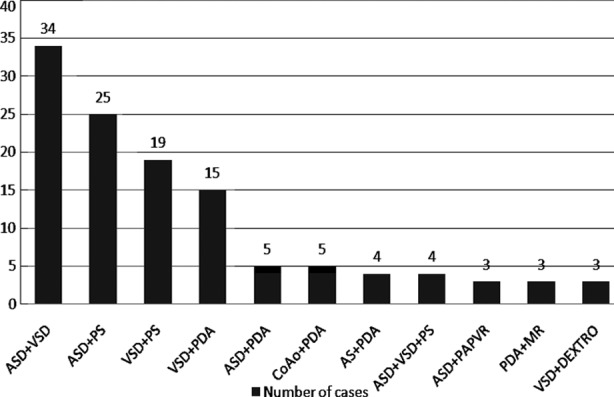
Cases of combinations.

In 147 (14.5%) cases combination of simple defects were encountered and the commonest combination was ASD with VSD in 34 cases followed by ASD with PS in 24 cases and VSD with PS in 19 cases.(Fig.1)

**Fig.2:**
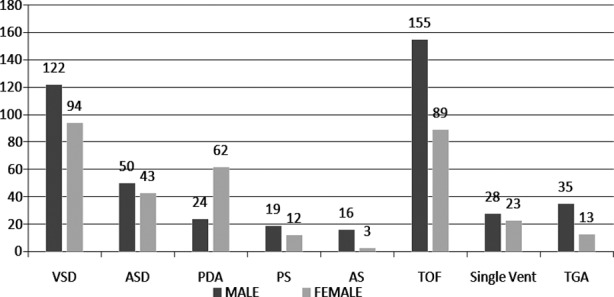
Gender Distribution of CHD.

The defects, ASD, VSD, PS, Aortic stenosis (AS) and MCHD were more prevalent in male participants and PDA was more common among females.(Fig.2) In 21 cases bicuspid aortic valve was present with other defects and in 18 cases dextro-cardia was found in combination with other defects.

## DISCUSSION

CHD was the most common congenital defect and had relatively higher mortality rate than other birth defects during first year of life. The incidence, prevalence and pattern of distribution of CHD types vary from region to region.^11^,^12^ In western industrial countries the incidence of CHD vary from 3-12/1000 live births.^13^ The meta-analysis report, which analyzed 114 studies from all over the world, showed that the highest birth prevalence of CHD 9.3/1000 live births is in Asia.^9^ WHO reports the incidence of CHD in Bangladesh is 6%, 15% in India, 6% in Burma and 10% in Sri-lanka.^14^

The studies from India report birth prevalence ranging from 3.9/1000^15^ to 26.4/1000^16^ live births. In our country, Pakistan, the only reported incidence is 4/1000 live births.^11^ According to the meta-analysis report the variation in the birth prevalence/ incidence is due to different methods of the studies analyzed and different diagnostic modalities used to detect the CHD. Moreover in developed countries the prenatal and natal examination detects even small defects like ASD which are asymptomatic.^9^ However the birth prevalence of 8-10/1000 live births is generally accepted worldwide and it is believed that it remained constant.^17^,^18^ This study was a hospital based study which included only cases of CHD which were registered in NICVD so it was not possible to find the prevalence or incidence. It shows the pattern of disease and frequency of defects. It can be understood that burden of CHD has increased significantly, from the fact that the targeted data was collected in just 20 days of Out-patient department.

The pattern of distribution of types of CHD encountered in this study was very different from other studies, whether conducted in our country or in other countries. The commonest type of CHD found in this study was TOF (245) 24.43% followed by VSD 21.5%, ASD 9.3% and PDA8.5%. While Study conducted at AKUH^11^, Lady reading hospital of Peshawar^19^ and at Hazara^20^ reported VSD as the commonest defect and the most common cyanotic defect was TOF.

In Hazara, out of 144 cases of CHD, 61.4% had VSD and relative frequency of TOF, ASD and PDA was 8.77%.^20^ The retrospective study conducted at Peshawar reported that out of 3072 cases of CHD 40.6% had VSD.^19^ The frequency of the CHD found in this study is compared with other studies of our country in the Table-III.

**Table-III:** Comparison of frequencies of Congenital Heart Defects with other studies of Pakistan.

Studies	Sadiq M^[21]^	Rahim F ^[22]^	Ahmad R et al ^[23]^	Aman W et al ^[19]^	Present study
VSD	1343(32.1%)	69 (46%)	180(42.2%)	1248(40.6%)	216(21.5%)
ASD	552(13.2%)	4 (2.6%)	56(14.08%)	493(16%)	93(9.3%)
PDA	536(12.8%)	4 (2.6%)	39(9.79%)	394(12.8%)	86(8.6%)
Pulmonary Stenosis	336(8.03%)	10(6.6%)	28(7%)	236(7.7%)	31(3.1%)
TOF	372(16.1%)	38(25.3%)	38(9.54%)	473(15.4%)	245(24.4%)
Number of patients	6620	150	398	3072	1003

In comparison with other countries of Asia, the same variation is encountered. The research from our neighboring country India reported that the most common lesion is VSD. In one study 21.3% cases of VSD were followed by ASD 18.9%, PDA 14.6% and TOF 4.6%.^16^ In another study 33% cases of VSD were recorded followed by ASD 19%, and TOF 16%.^15^ A study from Hajdu-Bihar reported a different pattern, the most frequent defect was ASD secundum followed by VSD and PDA.^24^

Similarly the age at which the initial diagnosis of CHD made was also different from other studies. In this study the majority of cases 74.7% were diagnosed in infancy and 59.3% cases have made their first visit to NICVD during 1^st^ year of life. The diagnosis in 75.43% of cases was made under one year of age, in the research conducted in Hazara.^20^ In Indian studies diagnosis was made in between zero and three years of age in 82.9% cases in one study^16^ and in other 58% cases were diagnosed in age group 0-5 years.^15^

Moreover in this study significant number of cases, 24.1% of combination of simple defects are recorded, among these the commonest combination is ASD with VSD 23.1% followed by ASD with PS 17%.

Such variation in frequencies of defects and the age of diagnosis, among this study and the other studies, may be because this study is conducted in a tertiary care cardiac hospital, NICVD, that receive referrals not only from all over the country but also from the neighboring countries like Iran and Afghanistan. The referred cases are usually those of MCHD that are diagnosed and referred at an early age because of the early appearance of signs and symptoms. This might explain the higher frequency of TOF and other cyanotic heart defects like Transposition of great arteries and single ventricle. Initial diagnosis of CHD types with respect to age is shown in Table-IV.

**Table-IV:** Diagnosis of CHD with respect to age.

Age Groups	CHD Types					

	VSD	ASD	PDA	PS	TOF	TGA
0-1 Year	181	42	56	18	200	45
>1-2 Years	7	10	11	8	17	2
>2-5 Years	16	16	13	4	18	1
>5-8 Years	4	3	3	0	8	0
>8-11 Years	2	7	0	0	1	0
>11-15 Years	5	5	1	1	0	0
>15-20 Years	1	2	0	0	1	0
>20-25 Years	0	2	2	0	0	0
>25 Years	0	6	0	0	0	0

Furthermore evidences support that variation in frequencies may occur due to the difference in detection methodology, provided facilities, variable genetic and environmental factors.^9^,^10^ A study conducted in Tibet reported that the CHD prevalence and composition differed significantly between populations of school children living above and below 4,200 m. In children group living at higher altitudes the prevalence of total CHD, PDA and ASD is higher than the children group living at lower altitudes, proving the environmental influence.^25^

Although the findings of this study are not consistent with the other studies but it proves the conclusion drawn in meta-analysis report that in Asia the prevalent CHD fall in the category of Pulmonary Outflow Obstruction and TOF.

## CONCLUSION

Congenital heart disease are very common in our setup and early detection rate of CHD is increasing. Moreover the general doctors and pediatrician diagnose CHD at an early age, and are becoming more aware of the complications of CHD that may develop if they refer late. Thus they are referring the CHD cases to cardiac centers for proper care at an early age. This increase in incidence may have some genetic basis. Besides these positive findings it cannot be ignored that burden of CHD is increasing and a proper population based study on a large scale is needed to estimate the prevalence accurately. In the meantime it is also needed to establish more cardiac centers that can provide proper and early treatment.

### Limitations of the study

Study was done only for short duration. Further study can be designed on this basis.
